# Despite Good Correlations, There Is No Exact Coincidence between Isometric and Dynamic Strength Measurements in Elite Youth Soccer Players

**DOI:** 10.3390/sports10110175

**Published:** 2022-11-10

**Authors:** Carl-M. Wagner, Konstantin Warneke, Christoph Bächer, Christian Liefke, Philipp Paintner, Larissa Kuhn, Torsten Brauner, Klaus Wirth, Michael Keiner

**Affiliations:** 1 Department of Training Science, German University of Health & Sport, 85737 Ismaning, Germany; 2 Department for Exercise, Sport and Health, Leuphana University, 21335 Lüneburg, Germany; 3 SSV Jahn Regensburg, 93059 Regensburg, Germany; 4 Institute of Health and Biomedical Innovation, Queensland University of Technology, Brisbane, QLD 4000, Australia; 5 Faculty of Training and Sports Science, University of Applied Science Wiener Neustadt, 2700 Vienna, Austria

**Keywords:** squat, maximal strength, 1RM, isometrics, jump, speed-strength, soccer

## Abstract

Speed strength performances are substantially dependent on maximum strength. Due to their importance, various methods have been utilized to measure maximum strength (e.g., isometric or dynamic) with discussed differences regarding transferability to sport-specific movements dependent upon the testing procedure. The aim of this study was to analyze whether maximum isometric force (MIF) during isometric back squats correlates with maximum strength measurements of the one repetition maximum (1RM) in the squat, with countermovement jump (CMJ) performance, and with drop jump (DJ) performances in elite youth soccer players (*n* = 16, 18.4 ± 1.5 [range: 17–23] years old). Additionally, concordance correlation coefficients (CCC, [*ρ_c_*]) between isometric and dynamic measurements were calculated to verify whether one measurement can actually reproduce the results of the other. To improve comprehension, differences between isometric and dynamic testing values were illustrated by providing differences between both testing conditions. For this, the mean absolute error (MAE) and the mean absolute percentage error (MAPE) were calculated. To reach equality in scale, the 1RM measures were multiplicated by 9.81 to obtain a value of N. The 1RM demonstrated correlations of τ = |0.38| to |0.52| with SJ and CMJ performances, while MIF demonstrated correlations of τ = |0.21| to |0.32|. However, the correlations of both 1RM and MIF with the DJ reactive strength index (RSI = jump height/contact time) from different falling heights were of no statistical significance. The data showed significant correlations between both the absolute (τ = |0.54|) and the relative (τ = |0.40|) performances of 1RM and MIF, which were confirmed by CCC of *ρ_c_*= |0.56| to |0.66|, respectively. Furthermore, the MAE and MAPE showed values of 2080.87 N and 67.4%, respectively. The data in this study show that, despite good correlations, there is no exact coincidence between isometric and dynamic strength measurements. Accordingly, both measurements may only represent an estimation of maximal strength capacity and cannot be substituted for each other. Therefore, maximal strength should be tested by using high similarity in the contraction condition, as it is used in the training process to counteract underestimation in strength because of unfamiliarity with the testing condition.

## 1. Introduction

Success in team sports, such as competitive soccer, is substantially dependent on superior speed strength performance (e.g., sprinting, jumping, rapidly performed directional changes) [1,2,3]. Speed strength actions contribute substantially to overall game performance and were reported with up to approx. 1300 speed strength actions per player per game [1,2,3]. Around 83% of successfully scored goals are preceded by sprinting, jumping, or rapidly performed directional changes [4]. Correspondingly, previous research identified differences in speed strength performances between varying age groups and performance levels (e.g., elite, sub-elite, amateur), suggesting that superior speed strength performance is associated with a higher level of soccer performance [5,6,7]. Superior speed strength performances require the generation of the largest possible ground reaction forces of up to five times body weight, which is accordingly highly dependent on maximum strength [8,9,10,11]. Thus, for youth soccer players striving to reach the highest levels of competition, it is mandatory to develop reasonably high levels of maximum strength in addition to other technical, tactical, and conditional qualities [1,3,5,12].

Due to its importance, various methods have been utilized to measure maximum strength (e.g., isometric or dynamic) with discussed differences regarding transferability to sport specific movements dependent on the testing procedure [13,14,15]. Squat jump (SJ) and countermovement jump (CMJ) performance showed correlation coefficients of r = |0.21| to |0.78| with dynamic free weight squats [16,17,18,19] and of r = |0.03| to |0.70| with isometric tests [19,20,21]. Correlations between dynamic free weight squats and drop jump (DJ) height have been reported with coefficients of r = |0.63| to |0.72| [22,23]. However, these studies did not report ground contact time, which is impeding the assessment of reactive strength ability [24]. Keiner, Kadlubowski, Hartmann, Stefer and Wirth [17] reported correlations between dynamic free weight squats and DJ reactive strength index (RSI = jump height/contact time) of r = |0.12| to |0.42|. Others reported correlations between DJ RSI and isometric strength, mostly measured via midthigh pull, of r = |0.3| to |0.54| [25,26]. Only Barnes, et al. [27] determined a correlation coefficient of r = |0.40| between isometric squats and DJ RSI. In general, the literature showed conflicting results regarding the influence of isometric and dynamic maximum strength on jumping performance, including studies with, in partial, small sample sizes [18], different testing procedures [17,25], and variant training levels of included participants [28,29].

Still, if both measurements of strength (dynamic and isometric) claim to describe the same basic concept of maximum strength, and be substituted for each other within performance diagnostic protocols, a high concordance must be assumed. However, none of the studies in the previous literature calculated concordance correlation coefficients between isometric and dynamic measurements to verify whether one measurement can actually reproduce the results of the other. Instead, relationships between maximal isometric forces (MIF) and dynamic one repetition maximum (1RM) measures generally range between to nearly perfect correlations (r = |0.52| to |0.97|) [30,31,32]. Furthermore, a limited number of studies and different study designs, e.g., different exercises (isometric mid-thigh pull vs. squat) impede conclusive results. Additionally, while both dynamic and isometric strength measurements are known as valid predictors of maximal strength with a good functional transfer to jumping and sprinting [33] concordance of both, maximum strength tests are potentially biased by testing modalities and their respective limitation (e.g., standardization of dynamic measurements, joint angle specificity and lack of familiarization with isometric measurements, and differences in motor unit recruitment and rate coding) which might further reduce correlation coefficients [13,14,34,35].

Therefore, the aim of this study was to analyze whether MIF during isometric back squats coincide with maximum strength measurements of the 1RM squat and how both measurements correlate with different jump performances (squat jump, countermovement jump, and drop jump), respectively. It was hypothesized that, while both isometric and dynamic squat strength would correlate with each other, correlations of 1RM squats to be higher with jump performances.

## 2. Materials and Methods

### 2.1. Experimental Approach to the Problem

**Figure 1:**
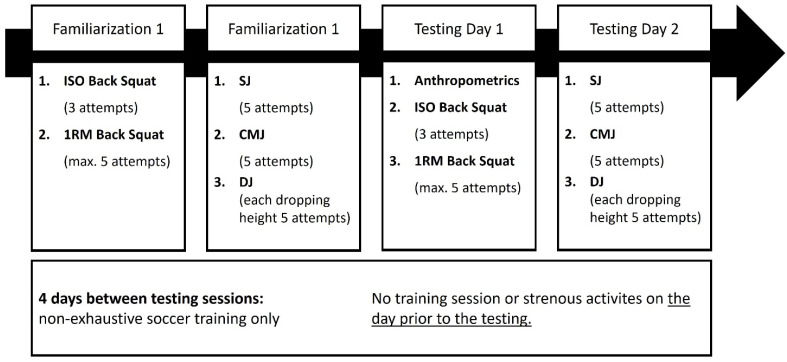
Study protocol.

To answer this research question, a cross-sectional study was conducted (see Figure 1). At the beginning of second half of the season (February 2022), 16 male youth soccer players from a youth elite training center were evaluated in their performances in SJ, CMJ, drop jump from 30 cm (DJ30), 45 cm (DJ45), 60 cm (DJ60) falling height, MIF, and 1RM in the squat to calculate relationships between strength and speed strength performances. One week prior to testing day 1, the soccer players completed familiarization sessions for all tests on two separate days. The familiarization sessions followed the same protocol as the actual test. The athletes were familiar with squats, SJ, and CMJ as part of their regular training routine. However, isometric strength testing and DJs weren’t part of their usual practice. The actual test protocol was divided into 2 testing days completed with 4 days rest between testing days. Maximum strength and speed strength measures taken on separate days. The 16 players were randomly divided into two groups. One group performed first the dynamic, then the isometric strength test on the testing day. The second group completed the strength tests in reverse order.

### 2.2. Subjects

Sixteen male youth soccer players (age: 18.4 ± 1.5 [range: 17–23] years old, height: 1.79 ± 0.06 m, body mass; 79.2 ± 7.0 kg) from the highest German youth league in their age group were analyzed. The soccer players were classified as elite in reference to the definition used by Lorenz et al. [36], who considered elite athletes as those who played at a higher level than peers within a sport. The sample consisted of 6 defenders, 5 midfield players, and 5 forwards, with a minimum of 10 years of training and competition experience in soccer. Their respective strength training experience ranged between 0 and 4 years. No goalkeepers were involved in the study. The players regularly performed 4 to 5 soccer sessions per week (8–10 h training/week). All subjects were regular starters and competed with their teams in their respective leagues on weekends during the season. The subjects did not participate in fatiguing training sessions for a minimum of 2 days prior to testing. None of the subjects reported any injuries at the time of testing. Each subject and their parents (for underaged participants) were informed about the aims of the study and the experimental risks involved with the research and provided written informed consent. Furthermore, this study was performed in accordance with the Helsinki Declaration and was approved by the Universities Ethics Committee (DHGS-EK-2021-002).

### 2.3. Procedure

On arrival, body mass (Seca Digital Scales, Model 707) and height (Stadiometer; Seca, Birmingham, UK) of all subjects were measured while in bare feet and wearing shorts.

The warm up for jump tests included nonspecific running with low-to-medium intensity for approximately 5 min, running with lifted knees, heeling, and side stepping. Afterwards, the athletes completed a 5-min dynamic stretching program (standing scales, hand walks, lunge steps with twisting and lateral lunges with rotation). Overall, the total warm-up time on each test day was 15 min.

First SJ, then CMJ were measured (5 trials each, with a 1-min rest between jumps) using a contact mat (Refitronic, Schmitten, Germany). The jumps were performed with the hands fixed on the hips. The SJ was initiated from a squat position (approx. 90° knee angle) after a 2-s hold without momentum. The CMJ was initiated from an upright position to utilize the momentum of a preceding squat movement (to approx. 90° knee angle) in the actual jump. On testing day, each subject performed 5 trials for each jump, with a 1-min rest between jumps, and the highest values obtained were registered for further analysis [19]. The test–retest reliability is reported between ICC = 0.96–98 [37]. 

The DJ test was carried out from 30 cm (DJ30), 45 cm (DJ45), 60 cm (DJ60) falling height. DJs were also measured (5 trials each) using the contact mat and self-manufactured boxes of corresponding heights. The subjects started with the height of 30 cm. With an initial step, subjects “fell” from a box (of corresponding height) and were instructed to jump as high as possible after both feet had contacted the ground. The hands were also fixed on the hips. They were further encouraged to reduce ground contact time to a minimum. Shorter durations of ground contact and higher jumps reflect better reactive power. RSI was calculated from these data (RSI = jump height/contact time). The participants paused for 1 min between jumps and 5 min between different jump heights. The highest values obtained within the five trials were registered for further analysis for each DJ height, respectively. The test–retest reliability of DJ is reported between ICC = 0.88–0.91 [17].

Testing included the determination of the 1RM for a back squat (high bar) in accordance with a standardized protocol [38]. The warm up for the strength measures consisted of three sets of back squats with progressive loads (40–70–85% 1RM) and a decreasing number of repetitions (approx. 6–8). The barbell (ATX Weightlifting training bar, ATX, Wassenberg, Germany) was positioned on the musculus trapezius pars descendens below the seventh cervical vertebra. The participants stood erect with a self-selected width of the feet, flexed their knees and hips to reach a deep squat position with proper form (top of thigh breaking parallel) and returned to the starting position. Based on the results of the familiarization session, the first attempt was performed with a load of approx. 90–95% 1RM. After successful attempts the load was increased by 2.5 to 5 kg. Attempts failed when the trained investigator (i.e., masters sport science, certified S&C coach with 15 years of experience) visually detected rounding of the back or insufficient squat depth. The determination of the 1RM was achieved within a maximum of 5 trials. The highest successful attempt was used for further analysis. The rest duration between attempts was 5 min. The test–retest reliability of 1RM for the squat is reported with ICC = 0.91 [17].

**Figure 2:**
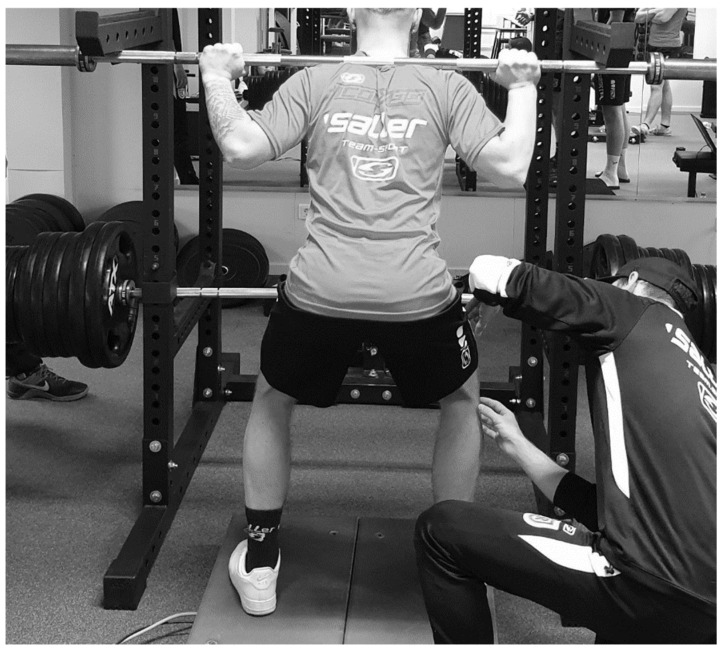
Setup for measurement of MIF in the isometric squat (120°).

MIF in each trial was recorded using two portable strain-gauge-based, one-dimensional (Fz) force plates (Twinplate, CONTEMPLAS GmbH, Kempten, Germany) (sampling rate: 1000 Hz) and plotted as force-time curves, indicating the peak force value. While standing in the squat rack, an empty barbell (20 kg) was positioned across the back of the participants. Participants were instructed to go into a standing squat position (high bar, knee angle of 120° and same foot position as during 1RM testing). Safety pins were placed in position to fixate the position of the barbell (Figure 2). Participants were instructed to generate a slight pretension against the safety pins and thereafter apply maximum pressure against the barbell and apply it for a period of three seconds. Subjects performed 3 attempts with a rest of 5 min between attempts. The joint angles were controlled via goniometer. A 120° knee angle was chosen, to use common joint angles used in the literature [33]. The highest values obtained within the three trials were registered for further analysis. The test-retest reliability of MIF for the squat is reported between ICC = 0.98–0.99 [39]. 

Relative force/strength values were calculated from force/strength performance divided by body weight [40]. 

### 2.4. Statistical Analysis

Calculations were performed using the statistical software package SPSS 27.0.1.0 (IBM, Ehningen, Germany). Descriptive statistics were calculated for all data and reported as mean ± standard deviations (SD) and 95% confidence intervals (95%CI). A Shapiro-Wilk test was performed to analyze the data for normal distribution. The best performances in each test were used for the statistical analysis. To assess the relative reliability of performances, ICCs and 95% CI were calculated from familiarization and testing sessions (inter-day reliability) and the 3 best performance of testing day (intra-day reliability) for squats and jumps. Relationships between the performance variables were calculated using one-tailed bivariate Kendall correlations (τ). The explained variance was calculated by squaring tau. To evaluate whether one method can reproduce the results based on another method, classic correlation coefficients (e.g., Pearson, Spearman, or Kendall) might not to be a valid method. If the first measurement is plotted against the second measurement and both measuring the same parameter, a 45° line through the origin would be estimated. Lin (1989) proposes the use of the concordance correlation coefficient (CCC, [*ρ_c_*]). To show the difference between Kendall’s tau correlation and to investigate whether both procedures measuring the same construct, CCC is determined in addition to tau (Version R ×64 4.1.3, Lucent Technologies. Dormagen, Germany). For this purpose, measured values were z-transformed by using $x_{i}\to \frac{x_{i}-{\bar{x}}_{n}}{\sigma }$. Because of differences in scale. To improve comprehension, differences between isometric and dynamic testing values were illustrated by providing differences between both testing conditions. For this, the mean absolute error (*MAE*) was calculated by using

$$MAE=\frac{1}{n}*\sum_{i=1}^{n}\left|x_{i}-y_{i}\right|$$

as well as the mean absolute percentage error (*MAPE*), which was calculated as follows

$$MAPE=\frac{100\%}{n}*\overset{n}{\underset{i=1}{\sum }}\left|\frac{x_{i}-y_{i}}{x_{i}}\right|$$

*MAE* and *MAPE* showed the error of the testing procedure with reference to the isometric value. For this purpose, and to reach equality in scale, the 1RM measures were multiplicated with 9.81 to obtain a value in N. Post-hoc power (1-ß) was calculated via G*Power 3.1.9.6 (University Düsseldorf, Düsseldorf, Germany).

## 3. Results

The descriptive statistics of the performance data are presented in Table 1. The Shapiro-Wilk test (*p* < 0.05) indicated a normal distribution for all measured variables except for the isometric force measurements. The data show high explained variance for both intra and interday reliability (Table 2). The intraday (test day) reliability is slightly higher than between the best values of the familiarization session and the test day (Table 2). The lowest reliability (interday) is shown for the variable DJ 60 with an explained variance of just over 70%.

The correlation coefficients are presented in Table 3 and Table 4. Post hoc power was calculated with 75% assuming an alpha level of 0.05 and considering a moderate effect size of r^2^ = |0.29|between 1RM and MIF. Dynamic strength performances showed correlation coefficients of τ = |0.42| and τ = |0.52| with SJ and CMJ heights, respectively, while isometric force showed correlation coefficients of τ = |0.23| to |0.32|, respectively. The confidence intervals overlap between the correlations of the jumping performances with the isometric force and dynamic strength performances, respectively. The absolute and relative strength performances showed similar correlation coefficients with the jump variables. None of the strength variables showed significant correlation with the RSIs from all drop heights. 

**Figure 3:**
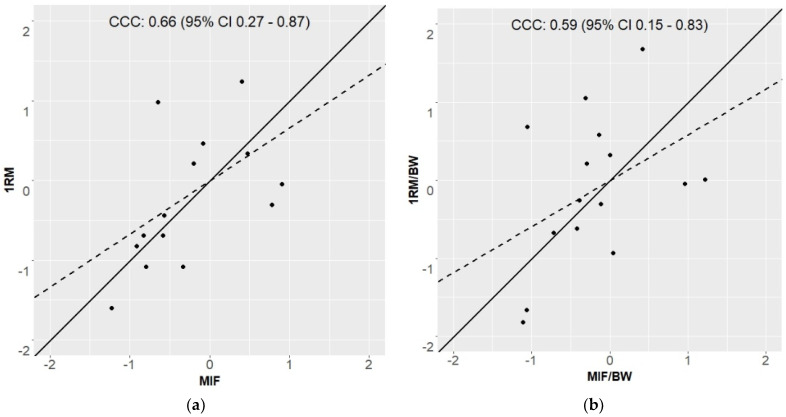
Scatter plot of standardized absolute (**a**) and relative (**b**) strength/force performances. 1RM = one repetition maximum; MIF = maximal isometric force; BW= body weight; CCC = concordance correlation coefficient (*ρ*_c_); 95%CI = 95% confidence interval; dashed line = 45° trend line; scattered line = linear trend line.

The absolute variables 1RM and MIF show a significant (*p* < 0.05) correlation of τ = |0.54|, the relative performances 1RM/BW and MIF/BW of τ = |0.40|, with an explanation of variance of 29% and 16%, respectively. The CCC was calculated for the absolute lines with *ρ_c_* = 0.66, and for the relative ones with *ρ_c_* = 0.56 (see Figure 3). These results confirm, with an explained variance of 31–43%, the results of the Kendall correlations. The absolute and relative data can be found plotted in Figure 2. Furthermore, the MAE and MAPE showed values of 2080.87 N and 67.4%, respectively.

## 4. Discussion

The study was designed to analyze whether the maximum strength measurements of isometric back squats correlate with the maximum strength measurements of the dynamic back squat and different jump performances among a population of elite male youth soccer players. The 1RM demonstrated correlations of τ = |0.38| to |0.52|) with SJ and CMJ performances, while MIF demonstrated correlations of τ = |0.21| to |0.32|. However, the correlations of both 1RM and MIF, with the DJ performances from different falling heights, were of no statistical significance. The study showed significant correlations between both the absolute (τ = |0.54|) and the relative (τ = |0.40|) performances of 1RM and MIF, which was confirmed by concordance correlation coefficients of *ρ_c_* = |0.56| to |0.66|, respectively.

In general, the results of this study corroborate previously reported correlations between speed strength performances, dynamic [16,17,19], and isometric [29,32] maximum strength measurements, respectively. In line with the findings of this study, Markovic and Jaric [32] previously reported correlations between MIFs during isometric back squats at 120° knee angle, squat jump, and countermovement jump height of r = |0.35| to |0.39| (*p* < 0.05). However, Loturco, Nakamura, Artioli, Kobal, Kitamura, Abad, Cruz, Romano, Pereira and Franchini [29] reported stronger correlations between MIFs during isometric back squats at 90° knee angle and dynamic jump performances (r = |0.79|, *p* < 0.01).

These findings support the idea that in order to increase the external validity of isometric strength testing methods to predict dynamic speed strength performances, the isometric joint angles should match the joint angles of the initial concentric force production during the dynamic movement [41], and could partially explain why this study failed to find significant relationships between isometric peak forces, squat jump, and countermovement jump performance, respectively. Variation in the magnitude of correlations between MIF and speed strength performances reported in various studies due to different knee angles might be argued by studies which directly investigated the effects of different joint angles during isometric strength assessments [39]. Based on the findings in the literature, most of the studies correlated speed strength performances with isometric squat strength at 90° and 120° knee angle, respectively [33,34]. The rationale behind these knee angles lies within the described maximal strength of the knee extensors at a joint angle of around 120° and the point with the worst leverage conditions (sticking point) at 90° [34]. Lum and Joseph [39] reported higher correlations between isometric squat strength at a 90° knee angle and CMJ performance (r = 0.43 to 0.56) than between isometric squat strength at a 120° knee angle and CMJ performance (r = 0.43). However, the literature directly comparing various knee angles during isometric strength testing is still scarce and Marcora and Miller [42] suggested the application of multijoint isometry at specific angles equally to the joint angles of highest power output during the corresponding dynamic movement to be more externally valid. However, these authors correlated SJ and CMJ with isometric measures of the leg press.

Furthermore, higher correlations of dynamic strength testing could be argued via task specificity in soccer players. As stated previously, dynamic as well as isometric strength testing can be seen as practical tools to estimate maximal strength. However, both dynamic and isometric testing procedures must be interpreted under consideration of their respective limitations. Accordingly, dynamic maximum strength may only be estimated, as the performance is limited by passing the point with the worst leverage conditions (sticking region), however there is still a movement [35]; while isometric maximum force, although it measures the maximum force against an insurmountable resistance at a certain joint angle, however, often also underestimates the true performance because mostly subjects are not accustomed to the contraction form [43]. From this, it can be hypothesized that only a high degree of task specificity (e.g., body position, joint angle, contraction mode, and movement pattern) accomplishes the necessary external validity to describe alterations in daily and sport-specific performances, which are primarily induced by dynamic training forms. Therefore, the external validity of isometric strength testing methods to predict dynamic speed strength performance has been questioned before and it is assumed that the reason for these discrepancies lie within structural, neural, and biomechanical differences [13,14,44]. Accordingly, it has been reported that structural differences within isometric and dynamic performance tests induce distinct movement patterns and contraction modes [15,45,46,47,48]. Consequentially, it has been argued that motor unit recruitment and rate coding may differ between isometric and dynamic muscle contraction [14]. Therefore, it was suggested that isometric and dynamic muscle actions must be understood as different physiological phenomenon [14] and maximum strength testing should occur preferentially in modalities specific to movement patterns of the athlete’s training and competition profile. However, in contrast, [31] pointed out very strong correlations between maximal isometric strength and dynamic movement tasks (squat, bench press, power clean) in elite wrestlers, in which a habituation of isometric muscle activity can be assumed. Consequently, one could speculate that maximal strength should be tested by using high similarity in the contraction condition, as it is used in the training process to counteract underestimation in strength because of unfamiliarity with the testing condition. From this, it could be recommended to use dynamic strength testing and avoid isometric strength testing, if the athlete’s training routine includes only a low level of isometric contractions, and vice versa. Regardless, calculation of CCC showed *ρ_c_* = |0.56| to |0.66|, together with qualitative analysis of Figure 2, strong differences regarding the estimation of maximal strength between both testing procedures can be assumed, which are confirmed by a MAE of 2080.87 N and a MAPE of 67.4%. These divergences seem to be not acceptable if it is hypothesized that both measurements examined the same ability.

With regard to the drop jump performances, the correlations of both 1RM (τ = |0.15| to |0.25|) and MIF (τ = |0.10| to |0.27|) with the various DJ performances were of no statistical significance. One of the reasons for this observation might be found in the structure of this test. Small knee flexion angles during ground contact and the reactive nature of the test, with ground contact times below 250 ms, facilitate only a limited length change in the quadriceps. Therefore, force generation by the calf muscles, as well as through elastic energy storage of elastic tissues and reflex mechanism, is required [12,49,50]. Consequently, when considering RSI by reducing ground contact time to a minimum, the muscles of the knee and hip joint might not influence DJ performance to the same extent as during SJ and CMJ tests. This is supported by significant correlations of 1RM standing calf raises relative to body mass with drop jumps from various heights (r = |0.36| to |0.57|) [17,51]. Furthermore, others reported a moderate influence of calf muscle strength on various jumping (r = |0.52| to |0.54|) and sprinting (r = |0.46| to |0.67|) performances [37,52]. Consequently, the influence of the calf muscles on drop jump performance implies their consideration in athletic training regimes and diagnostic protocols [17].

This study has potential limitations. Based on the study design investigating a soccer team with a fixed number of members, an a priori sample size estimation via G*Power was not feasible. Accordingly, the relatively small sample size resulted in a broad range of 95% CI. Therefore, the differences between the various correlation coefficients (see 95% CI) must be interpreted with caution. Still, based on the post hoc G*Power analysis the sample size is sufficient to allow a solid (Power [1-β err prob] 75%) evaluation of the relationships between the analyzed variables. Furthermore, the investigated sample consisted of multiple field positions which could imply differences in performance levels and therefore affect correlations. However, all players received the same training routines over the years and no goalkeeper was included in the sample. While this study used common protocols for the performance test [33], the different tests within this study were performed in different joint angles partially (e.g., 90° vs 120°). This might have reduced the correlation coefficient between the different performances, as it was suggested that the isometric joint angles should match the joint angles of the initial concentric force production during the dynamic movement [41]. Therefore, MIF at a knee angle of 90° potentially would have led to lower strength values, however higher correlations with dynamic maximum strength and speed strength measures [53]. Despite these limitations, the results of this study are valuable since the literature comparing isometric and dynamic strength measurements without a structural bias is scarce. 

## 5. Conclusions

The data of this study show that, despite good correlations, there is no exact coincidence between isometric and dynamic strength measurements. Accordingly, both measurements may only represent an estimation of maximal strength capacity and cannot be substituted for each other. Therefore, maximal strength should be tested by using high similarity in the contraction condition, as it is used in training process to counteract underestimation in strength, because of unfamiliarity with the testing condition. Therefore, assessing maximum strength via the 1RM parallel squat within soccer specific performance diagnostic protocols might provide more relevant information than isometric strength measures about the athlete’s maximal strength abilities, as the athletes are more familiar with the movement patterns within this testing modality. 

## Figures and Tables

**Table 1:** Descriptive statistics of performance variables.

	Mean ± SD	95%CI
MIF (N)	3071 ± 647	2754–3388
MIF/BW (N/kg)	40.3 ± 7.9	36.4–44.2
1RM (kg)	100.9 ± 20.0	91.1–110.7
1RM/BW (kg/kg)	1.31 ± 0.20	1.21–1.41
SJ, height (cm)	37.6 ± 4.0	35.6–39.6
CMJ, height (cm)	39.4 ± 4.2	37.3–41.5
DJ30, height (cm)	33.7 ± 5.6	30.7–36.7
DJ30, contact (ms)	186 ± 18	176–195
DJ30, RSI	1.84 ± 0.41	1.62–2.06
DJ45, height (cm)	33.8 ± 6.0	30.6–37.0
DJ45, contact (ms)	184 ± 22	173–196
DJ45, RSI	1.87 ± 0.47	1.62–2.12
DJ60, height (cm)	33.7 ± 7.2	29.8–37.5
DJ60, contact (ms)	192 ± 24	180–205
DJ60, RSI	1.86 ± 0.51	1.60–2.14

SD = standard deviation; 95%CI = 95% confidence interval; MIF = maximal isometric force; 1RM = one-repetition maximum; SJ = squat jump; CMJ = countermovement jump; DJ30,45,60 = drop jump from 30 cm, 45 cm, 60 cm falling height; height = jump height; contact= ground contact time; RSI = reactive strength index; N = Newton; BW = body weight; kg = kilogram; cm = centimeter; ms= milliseconds.

**Table 2:** Reliability analysis of the performance variables.

	Intra-Day Reliability		Inter-Day Reliability	
	ICC	95%CI	ICC	95%CI
**MIF**	0.97	0.91–0.99	0.97	0.90–0.99
**1RM**	only one maximal attempt per day		0.98	0.93–0.99
**SJ**	0.99	0.97–0.99	0.93	0.80–0.98
**CMJ**	0.97	0.92–0.99	0.91	0.74–0.97
**DJ30**	0.98	0.96–0.99	0.95	0.84–0.98
**DJ45**	0.98	0.96–0.99	0.94	0.81–0.98
**DJ60**	0.97	0.93–9.99	0.84	0.53–0.94

ICC = intraclass correlation coefficient; 95%CI = 95% confidence interval; MIF = maximal isometric force; 1RM = one-repetition maximum; SJ = squat jump, CMJ = countermovement jump; DJ30,45,60 = drop jump from 30 cm, 45 cm, 60 cm falling height.

**Table 3:** Kendall’s tau with 95% confidence interval between absolute strength/force and jump performance.

	**SJ**	**CMJ**	**DJ30**	**DJ45**	**DJ60**
**1RM**	0.42 * (−0.03–0.87)	0.52 * (0.12–0.92)	0.20 (−0.32–0.72)	0.22 (−0.30–0.74)	0.20 (−0.32–0.72)
**MIF**	0.23 (−0.29–0.75)	0.32 * (−0.17–0.81)	0.27 (−0.23–0.77)	0.22 (−0.30–0.74)	0.10 (−0.44–0.64)

1RM = one-repetition maximum; SJ = squat jump, CMJ = countermovement jump; DJ30,45,60 = drop jump from 30 cm, 45 cm, 60 cm falling height; * = *p* < 0.05.

**Table 4:** Kendall’s tau with 95% confidence interval between relative strength/force and jump performance.

	**SJ**	**CMJ**	**DJ30**	**DJ45**	**DJ60**
**1RM/BW**	0.38 * (−0.09–0.85)	0.50 * (0.09–0.91)	0.15 (−0.38–0.68)	0.17 (−0.36–0.90)	0.25 (−0.26–0.76)
**MIF/BW**	0.21 (−0.31–0.73)	0.22 (−0.30–0.74)	0.15 (−0.38–0.68)	0.13 (0.40–0.66)	0.18 (0.35–0.71)

1RM = one-repetition maximum; SJ = squat jump, CMJ = countermovement jump; DJ30,45,60 = drop jump from 30 cm, 45 cm, 60 cm falling height; BW = body weight; * = *p* < 0.05.

## Data Availability

Data are available upon reasonable request.
